# Augmented Robotics Dialog System for Enhancing Human–Robot Interaction

**DOI:** 10.3390/s150715799

**Published:** 2015-07-03

**Authors:** Fernando Alonso-Martín, Aívaro Castro-González, Francisco Javier Fernandez de Gorostiza Luengo, Miguel Ángel Salichs

**Affiliations:** Robotics Lab, Universidad Carlos III de Madrid, Av. de la Universidad 30, Leganés, Madrid 28911, Spain; E-Mails: acgonzal@ing.uc3m.es (A.C.-G.); jgorosti@ing.uc3m.es (F.J.F.G.L.); salichs@ing.uc3m.es (M.A.S.)

**Keywords:** augmented dialog, augmented interaction, contextualized dialog, social robots, human–robot interaction, HRI, natural language understanding, natural language processing, interaction system, dialog system, multimodal interaction

## Abstract

Augmented reality, augmented television and second screen are cutting edge technologies that provide end users extra and enhanced information related to certain events in real time. This enriched information helps users better understand such events, at the same time providing a more satisfactory experience. In the present paper, we apply this main idea to human–robot interaction (HRI), to how users and robots interchange information. The ultimate goal of this paper is to improve the quality of HRI, developing a new dialog manager system that incorporates enriched information from the semantic web. This work presents the augmented robotic dialog system (ARDS), which uses natural language understanding mechanisms to provide two features: (i) a non-grammar multimodal input (verbal and/or written) text; and (ii) a contextualization of the information conveyed in the interaction. This contextualization is achieved by information enrichment techniques that link the extracted information from the dialog with extra information about the world available in semantic knowledge bases. This enriched or contextualized information (information enrichment, semantic enhancement or contextualized information are used interchangeably in the rest of this paper) offers many possibilities in terms of HRI. For instance, it can enhance the robot's pro-activeness during a human–robot dialog (the enriched information can be used to propose new topics during the dialog, while ensuring a coherent interaction). Another possibility is to display additional multimedia content related to the enriched information on a visual device. This paper describes the ARDS and shows a proof of concept of its applications.

## 1. Introduction

The area of human–robot interaction (HRI) is devoted to investigating the relations between robots and humans and how they communicate. The main long-term aim is to allow a natural interaction between humans and robots in ways that mimic human–human communication.

In the last decade, dialog systems (in the context of this paper, we consider dialogs as a bidirectional flow of messages or information, using many possible communicative modes, such as verbal and non-verbal language, between two or more agents; therefore, “dialog” and “interaction systems” might be considered equivalent terms) began to consider several ways or channels to share a message between interactors [1–5]. These channels are called modalities, or simply modes. For instance, verbal utterance, written information, touching events or gestures are different modes that can be used as inputs or outputs during a dialog. When a dialog employs several input or output modes, it allows a multimodal interaction, which is an essential feature of natural interaction [6]. In social robotics, the most popular input mode is the voice, which is processed by automatic speech recognition systems (ASR), and the most popular output mode is a verbal robot utterance, usually generated by a voice synthesizer (text-to-speech system (TtS)). Although voice mode is the most used, it is usually accompanied by gestures, such as pointing, deictic gestures or gaze.

On the other hand, the HRI community is increasing its interest in the application of social robots with patients, the elderly or children [7–14]. These groups require, for their care, a high level of attention and dedication by both caregivers and relatives. Social robots might certainly offer great benefits in their use as partners and collaborators with caregivers, relatives and patients. However, usually, these groups have severe difficulties in communicating with robots [15], and these scenarios are full of numerous challenges. When robots interact with persons from these groups, the interaction is often strictly predefined by the designer/programmer, and the communicative messages that the robot might understand are very limited. Due to this communicative limitation and the cognitive problems associated with these groups (lack of attention, difficulties in reasoning, lack of memory or language comprehension), the communication can fail, and consequently, the HRI can be felt as unsatisfactory [16]. This is one of the reasons that prevents social robots from moving out of the lab and into houses, hospitals, schools or other public domains. We believe that the application of multimodal dialog systems to HRI with social robots will enhance the interaction and, so, the acceptance of these robots in society and, in particular, by these groups of people with special difficulties.

One of the big limitations that we can find in HRI is the lack of capacity to extract semantic and relevant information from the user's utterance. On the other hand, social robots, as electronic devices, may offer new features, such as access to the web and the Internet, and new expression modalities, such as the control of other external electronic devices, such as an external screen.

We conceive of social robots as conversational agents with a physical body living in a dynamic environment. If we want them to succeed in these dynamic environments, they have to adapt the dialogs to changes in the environment, *i.e.*, the dialogs have to be contextualized.

We consider that a dialog is contextualized when the references to the environment are resolved. In order to achieve a proper contextualization, it is necessary to have some type of knowledge of the external environment (the world the robot is living in) and of the robot itself (its own history of previous interactions or its current state). A contextualized dialog includes situated dialog [17], that is the robot has to be able to understand sentences like “turn to my right” (it needs to know the pose of the user) or “it's the second room when passing the lab” (it needs to know where the lab is). These sentences can be considered grounded or understood if the robot has models of the environment (a map where the lab is annotated) and the user (a user tracking system providing the user's pose). Moreover, contextualized dialogs have to be able to adapt to unexpected content. For example, if a person says “yesterday, I watched the game between Real Madrid and Barcelona”, a contextualized dialog has to be able to extract enriched information from that utterance, e.g., Real Madrid is a football team from Madrid, Spain. Such enriched information can be used to provide data about the game or the teams.

The augmented robotic dialog system (ARDS) presented in this paper tries to contribute to improving human–robot dialogs, resolving some semantic reference problems involved in perception and incorporating new modalities into the expression system.

The rest of this paper is organized as follows. In Section 2, we give a synopsis of how the information flows between the developed components. Here, the robotic platform is also shown. In Section 3, we explain how linguistic theories about language are applied to the proposed system. In Section 4, the robotic dialog system is presented as the previous system that is used as the basis for this work. In Section 5, we introduce the new components that form the augmented robotic dialog system (ARDS) and its new features. We then present the perception components: optical character recognition (OCR) and automatic speech recognition (ASR). Moreover, the information enrichment process is described. In Section 6, we show a proof of concept, where a person and a robot are having a conversation, and by means of the ARDS, the robot complements the topic of the conversation by displaying web content on an external screen. Finally, some conclusions and lines of future research are presented in Section 7.

## 2. System Overview

**Figure 1:**
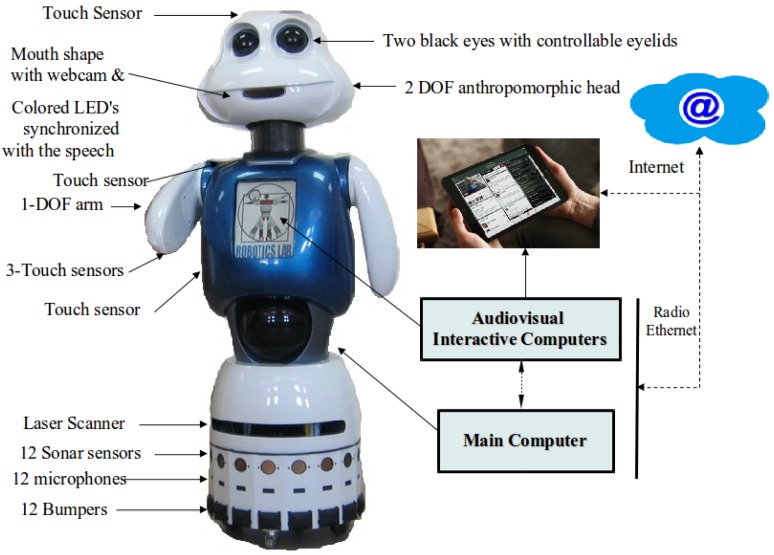
The social robot Maggie with an external interactive tablet.

Figure 1 shows the social robot Maggie with some details of the hardware and an external tactile tablet that is accessible to the robot. Maggie, as a robotic research platform, has been described with more detail in other papers ([6,18], and many others), and here, we shall focus on those components relevant to the present paper.

The web cam in the mouth allows using computer vision algorithms. For speech recognition, we use remote SingStar[19] wireless microphones. One main computer takes charge of the robot's main control. There is a tactile computer for screen interaction in the body of the robot, and other external tactile devices are also accessible both as input (tactile) and output (visual) interfaces.

In robotics, a dialog system is intended to ease the communication between users and the robot [20]. The ARDS is based on a previous multimodal dialog system, the robotics dialog system (RDS), whose components have been presented in several papers [21–24]. In the present paper, the RDS is extended with two additional features: (i) non-grammar-based input modalities, both verbal and written; and (ii) the addition as outcomes of extra visual information related to the context, topic and the semantic content of the dialog itself.

The first functionality (non-grammar-based inputs) makes the interaction more natural and easier than with a grammar-based system. It is not limited by the rules of a grammar (a list of words and their combinations), but it is open to any word in any position. We provide two input modules for this feature: one in charge of the non-grammar-based ASR and the other for the optical character recognition (OCR).

## 3. Natural Language Levels in the System

The research presented in this paper comprises several areas: dialog manager systems (DMS), automatic speech recognition (ASR), optical character recognition (OCR), information extraction (IEx) and information enrichment (IEn). We have been inspired by several papers from these areas and have taken available tools for doing a whole integration in a real interactive social robot. How the system should get the semantically-relevant information from the user and how it should show the enriched information are based on the layers in which a natural language is structured.

In order to achieve the features outlined, we need to develop systems able to achieve a high level of natural language understanding (NLU) following the strategies depicted in [25]. For instance, in the second chapter, four main techniques were proposed for realistic language comprehension (RLC) systems in regard to translating “open” and natural inputs to high-level knowledge structures, modifying these results to fit domain-based reasoning rules [26]. Furthermore, Liddy [27] and Feldman [28], based on the levels of Morris [29], suggested that in order to understand natural languages, it is important to distinguish between seven interdependent levels.

1. Symbolic level: This comprises the atomic indivisible signs that are combined to produce larger symbols. If the language is verbal, this corresponds to the phonological level and deals with the pronunciation of the words as speech sounds or phonemes. For written text, these signs are just letters.
2. Morphological level: This refers to the minimum meaningful unit, *i.e.*, lexemes, prefix and suffixes.
3. Lexical level: This level is related to the vocabulary of the language used. Each word is categorized according to its function in the sentence (noun, adjective, verb, *etc.*).
4. Syntactic level: This level deals with the grammatical structure of the sentences. It identifies subjects, predicates, direct and indirect objects or nuclei.
5. Semantic level: This is linked to the meaning of the words and sentences, that is for which points they are relevant to the person.
6. Discourse level: This refers to the message conveyed in a set of sentences. For a verbal language, this level deals with the meaning of the whole speech; in the case of a written language, it considers paragraphs.
7. Pragmatic level: At this level, the meaning of the discourse is enriched with additional information (not explicitly included in the sentences) coming from the environment, the world or the culture.

Here, we adapt this scheme to our framework on how common people usually extract meaning from a text or spoken language. If we consider a person saying “Yesterday, I went to Bernabeu Stadium to see the Real Madrid-Barcelona match”, in the phonetic or phonological level, we could find phonemes, such as in the word yesterday, (phonetic transcription of yerterday is: *'yεstə′,der*), that are very much related to the spoken language; at the lexical level, words, such as the pronoun “I”, the noun “stadium” or the verb “went” (the past tense form of “to go”), are organized in a syntactic structure where the verb follows the subject. The syntactic level deals with the grammatical structure of the sentences, so with the correct grammatical sentence structures. Automatic speech recognition and optical character recognition techniques use this grammatical information to improve the accuracy of the transcription of the sentences; for this, they use statistical language models or handwritten grammars (for detailed information about ASR techniques in robotics, see [30]). In this sense, ASR and OCR techniques cover these levels: symbolic, morphological, lexical and syntactic.

However, the most relevant levels for our system are the semantic, discourse and pragmatic levels. The semantic level is related to the deep syntactic structure and can only be understood by someone who has an idea or experience about a soccer game, a stadium and the related teams. The discourse level is where it makes sense why the person asserts that he/she went to such a game. The pragmatic level is directly related to the consequences for the environment and others of having expressed that information. For instance, this information could cause curiosity about which team won, whether the game was entertaining or with whom the person was.

The proposed system, ARDS, has been developed using some NLU techniques that work at different language levels matching the structure described above. In what follows, we describe the NLU techniques and how they are applied to the language levels.

1. Optical character recognition (OCR): This digitizes written text. This technique makes the written communicative mode accessible to the interaction system. This mode can be complementary to the verbal mode, and both can be used as inputs at the same time. For example, a person and a robot can talk about a certain written text where the person wants to know how to pronounce a word (not written in their native language) or just wants more details about a proper noun: “Tell me what you know about this” while showing the written proper noun.
2. Automatic speech recognition (ASR): We use a statistical language model (SLM) [31], a non-grammar-based ASR to translate open natural speech into text, which is akin to a speech dictation tool.
3. Information extraction (IEx): Considering that ASR and OCR do not provide processed information, at this level, some meaning of the message is extracted and expressed in a high-level language. IEx techniques analyze the digital texts provided by ASR and OCR and produce processed information, e.g., the entities, concepts, topic or user sentiment.
4. Information enrichment (IEn): This process refers to the extension of a message with additional information. It is related to the pragmatic level, where new and external information is added. This new information is obtained from knowledge bases that structure and classify information from the world. There are several free online services that provide access to these knowledge bases. Information Enrichment is also referred to by other authors as contextualized information, enriched information, semantic enhancement, enhanced information or augmented information.

## 4. The Robotics Dialog System: The Framework for the Augmented Robot Dialog System

**Figure 2:**
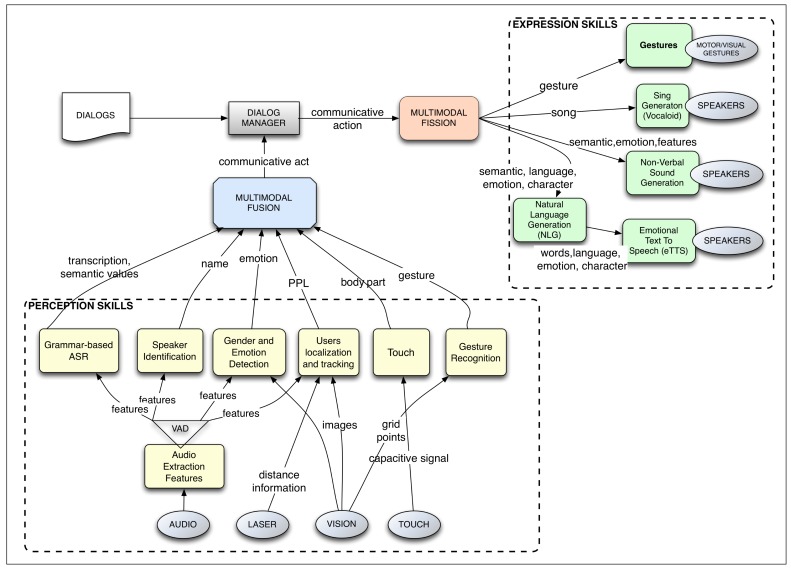
Sketch of the main components of the robotic dialog system: perception skills that feed the multimodal fusion module, the dialog manager, the multimodal fission module and the expression skills that control the actuators (hardware elements).

As mentioned before, the ARDS is an extension of a previous dialog management system, called the robotic dialog system (RDS). Here, we briefly describe the RDS to facilitate understanding of the rest of this paper. More details can be found in [22–24,32]. The RDS is intended to manage the interaction between a robot and one or more users. It is integrated within the robot's control architecture, which manages all of the tasks and processes, which include navigation, user interaction and other robot skills. See Figure 2.

Summarizing, the RDS works as follows:

1. Sensory perception obtains raw data from the world in different modalities. For instance, in the visual mode, it uses an RGB camera, a Kinect or a laser telemeter; in auditive mode, it uses microphones; in tactile mode, it uses capacitive sensors and RFID readers.
2. Perception skills process the raw data. They produce higher level information, transforming the raw data into more meaningful data. For example, when a user is talking to the robot and touches the robot's arm, the ASR translates the user's speech to text, and the tactile skill determines that the left arm has been touched. In the RDS, the ASR skill uses semantic context-free grammars (CFGs) to obtain such relevant information.
3. Multimodal fusion: The incoming information from the perception skills is fused in packages of semantic values within different time frames [24]. For instance, if the user says “Raise it” while touching the robot's left arm, the multimodal fusion module receives separately the user's sentence and the body part that has been touched and fuses them, concluding that the user wants the robot to raise its left arm.
4. Dialog manager: The output from the fusion module is taken as input by the dialog manager (DM), which handles the interactive skills and the state of the dialog. The multimodal information triggers the transitions that shape the flow of the dialog. Dialogs are defined as fixed plans where a multimodal input changes the state of the plan; the goal of the plan is to fill a set of information slots. The DM selects which action to take and how and when to execute it. The output of the DM represents the necessity to communicate a message, but the chosen action can be non-communicative. For instance, if the RDS is running a dialog for teleoperating the robot by voice, once the DM receives the message in advance, it sends out the instruction to do it, expresses a confirmation message and returns to an idle state waiting for new inputs.
5. Multimodal fission: This module takes the expressive instructions received by the DM and defines how to articulate the communicative expression towards the environment, controlling which output communicative modes have to be used and how. For instance, for executing a multimodal gesture, the module might decide to send the command for raising the arm to the module controlling the movements of the arms and another command to the voice skill in order to synthesize an informative message about the action.
6. Expressive skills endow the robot with the ability to communicate using different modes. Each mode is controlled by a skill that is connected to the hardware, that is the actuators. For instance, the emotional text-to-speech (eTtS) skill for verbal communication, the skill for controlling the robot's gestures and poses or the skill of controlling the color of the robot's cheeks. Continuing with the previous example, a command to raise the right arm would be translated by the pose skill into “move the motor with id LEFT_ARMto 145 degrees”, while the eTtS skill synthesizes the sentence “OK, I'm raising my left arm”.
7. Actuators are controlled by the expressive skill. Actuators, as hardware devices, execute low-level commands. Examples of these actuators are sound cards, motors, servos or LEDs.

Figure 2 shows the components of the RDS. It is important to note that the different modules run concurrently and in a general control loop, so the robot is perceiving, fusing information, moving the dialog flow and acting, all at the same time. This system is the framework where the ARDS has been developed.

## 5. The Augmented Robotic Dialog System

**Figure 3:**
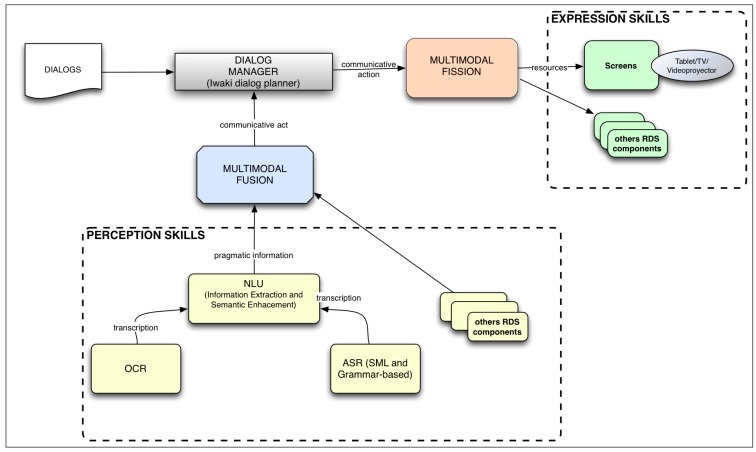
Sketch of the augmented robotic dialog system (ARDS). The system includes new components not in the RDS, information extraction and information enrichment, and a new component for managing a screen as a communicative mode. Below the text recognition component (OCR) and the speech recognition (ASR) component are represented. ASR can work in two different modes: a statistical language model (no grammatical restrictions) or a grammar-based mode.

Figure 3 depicts the different components of the ARDS. The new input functionality is presented as the following input modules: OCR and non-grammar ASR. The other new functionality is presented in the NLU module. This component takes the information extracted from the OCR and ASR modules and extracts some of its semantic content. Another new component finds, from some knowledge bases, related enriched information, such as pictures, videos and related links, which is fused into multimodal input information and sent to the component responsible for managing the dialog flow, the DMS. The current dialog may use that enriched information to express a multimodal message that includes it.

### 5.1. Introduction to the ARDS

**Figure 4:**
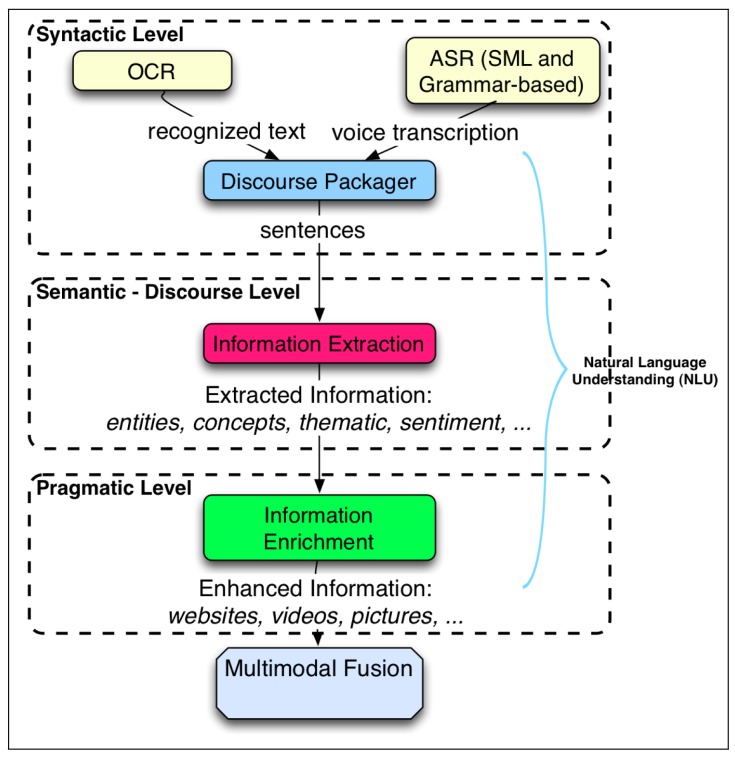
General overview of the information flow inside the augmented robotic dialog system.

The ARDS proposed in this paper extends the RDS with new features. Figure 4 depicts how the ARDS works. Initially, at the top of the figure, the user's utterances are transcribed into an electronic format. These phrases can be verbal, written or a mixture of both. These transcriptions correspond to the syntactic layer of the natural language layers explained above, since they involve the grammatical structure of the sentences.

Then, considering a certain discourse frame time (fixed in the discourse packager module, so as to get an appropriate trade-off between the promptness of the results and the right contextualization of the information), these utterances are grouped by the discourse packager. The resulting sentence is processed by the information extraction module, which pulls out important processed information: a set of entities and concepts, their categories, the dominating sentiment of the sentence and expressions related to time, phone numbers and to currency. This information corresponds to the discourse level of the natural language layers.

The set of entities and concepts are sent to the information enrichment module. Here, this information is enriched by adding related additional information. This information corresponds to the pragmatic level of the natural language layers.

All of these data, the extracted information and the enhanced information, are passed to the multimodal fusion module, where it is merged with the information coming from the other perception skills. The rest of the process is as in the RDS explained above.

### 5.2. Perceptual Components in the ARDS

#### 5.2.1. Unrestricted Verbal Input

In the area of ASR and dialog management, Bellegarda presented a statistical ASR system and a dialog manager (Siri) to perform natural interactions between a smart-phone and a user [33]. This system is intended to work as a personal assistant. Other systems, such as that of Yecaris *et al.* [34], use statistical approaches for automatic speech recognition. However, most speech recognition systems use context-free grammars (CFG) for facilitating the search process.

The main advantage of using grammars such as the CFG used by the RDS is that the system can easily constrain the verbal information that is relevant for the robot, which also increases the recognition accuracy. The main disadvantage is that the system is not able to recognize user utterances that are not represented by the defined CFG.

The ADRS adds a grammarless interaction, allowing a user to communicate with the robot without such grammatical restrictions. This means that there is no set of rules that defines the possible communicative options. To this end, we applied: (i) ASR tools using statistical models of the language to transcribe the user's speech; (ii) OCR for written communication; and (iii) information extraction and enrichment techniques to obtain extra information for the dialog.

The former element translates spoken words into text without using grammars as predefined rules. We employ the Google ASR web service (Version 2.0) [35] for translating the user's utterances. Alternatively, we have used other cloud ASR services, such as Nuance ASR and AT&T). Before we call this service, we need to distinguish between the voice and other noises and to clearly identify when the utterance starts and ends. For this purpose, we used the voice activity detector system (VAD) developed by the authors and presented in [23]. The VAD system is also integrated in ADRS, so the utterance is finally recorded in a file that is sent to the Google ASR service, which returns the translated written text.

The timing performance of this module depends on the communication bandwidth. During our experiments, the average response time from the end of the utterance to the delivery of the translated text was around 1.5 s.

#### 5.2.2. Optical Character Recognition

**Figure 5:**
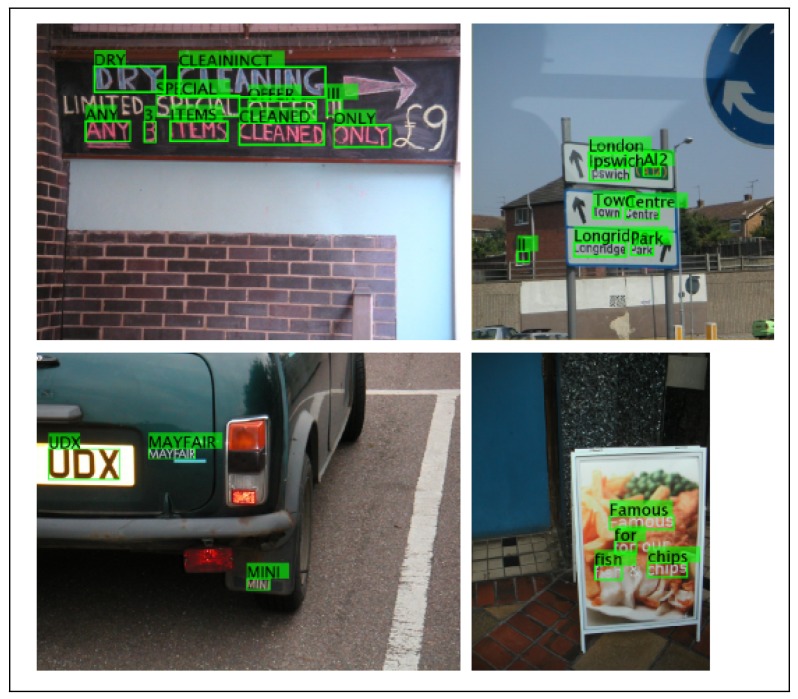
Optical character recognition in real time.

The technique of OCR has been extensively applied. However, only a few researchers have worked on real-time OCR. Milyaev *et al.* [36] recognized text from a real-time video (see Figure 5). The text can be written on different surfaces, and its size, position and orientation can vary.

On the other hand, the OCR module allows the interaction with the robot to use written text. It digitizes handwritten or printed text; thus, these can be used by a computer. We use the free tool called Tesseract OCR, also from Google [37]. This software runs locally in the robot, and the processing time is between 0.5 and 2 s, depending on computer performance.

Both the open grammar ASR and the OCR work concurrently and send the recognized text to the discourse packager module.

#### 5.2.3. The Discourse Packager

This module concatenates the outputs from the ASR and the OCR modules into one sentence using a period as the time frame delimiter. The size of the resulting sentence can be determined by this time frame or by the number of sentences that have to be gathered.

In case we want more frequent inputs to the dialog, we do not concatenate the phrases; they are forwarded as they arrive. This implies less information conveyed in the sentence. In case we want to better understand the context and reduce any potential ambiguity in future steps, we should consider concatenating phrases, defining a time frame or a sentence size.

However, if the time frame or the size is too big, this leads to delays in the analysis of the sentence and, consequently, in the transitions of the dialog, which leads to a careful balance between the dialog speed and its precision or accuracy. The explained parameters for the discourse packager have been chosen empirically by trial-and-error experiments. An automatic approach to such adjustment would be an appealing issue for future development.

### 5.3. The Enriched Information in ARDS

#### 5.3.1. Information Extraction

Regarding the IEx issue, Balchandran's patent [38] describes the main phases that are necessary to obtain semantic information from a general plain text. Furthermore, Xu *et al.* [39] proposed a system to automatically generate tags based on the contents of social webs. Using IEx mechanisms, this system generates tags that are known as folksonomies, *i.e.*, tags based on social media content. Another tagging system was presented by Choi *et al.* [40]. They state that their contributions are (1) to systematically examine the available public algorithms' application to tag-based folksonomies and (2) to propose a service architecture that can provide these algorithms as online capabilities.

In general, IEx refers to the task of analyzing a text and classifying its information. For example, there are methods to obtain the key words within a text, the main mood of the discourse or the gender of a term. Each one of these methods is an active research field by itself. These methods extract semantic information from the sentences. Consequently, they correspond to the semantic and discourse levels within the natural language structure described above.

The IEx techniques used in this paper are based on web services: Textalytics/MeaningCloud [41], Semantria [42], Bitext [43] and Lextalytics [44]. These are paid services that offer a free data quota. The IEx module can use all of them, but only one at a time, so the information coming from the different services is not combined. Internally, these services run different algorithms, and all of them provide the same extracted data:

- Sentiment: This refers to the mood or tone of the message, which is computed by analyzing the affective aspects of the words within the sentence. In short, when there are many compliments, the sentiment is positive; if, for instance, insults or scorn are plentiful in the sentence, the sentiment would be negative. Otherwise, the sentiment will be neutral.
- Entities: This technique obtains the proper nouns (people, places, items, organizations, *etc.*). Usually, huge specialized dictionaries are used for each one of the possible types. The performance of this technique depends on its complexity. Searching for words starting with a capital letter is not enough (the first word in each sentence starts with a capital letter, too, but this does not imply that it is a proper noun); or some words, or group of words, can be ambiguous (they can refer to more than one type of entity).
- Concepts: In this case, algorithms that analyze the grammatical structure of a text find common nouns as concepts, e.g., “ball”, “building” or “table”.
- Theme: This is a topic that is more related to the input sentence. There are many possible topics, and the IEx module gives the closest one, e.g., “politics”, “sports” or “art”.
- Time expressions: This identifies the adverb of time (e.g., yesterday afternoon, tomorrow or the day before yesterday) and completes these data with these adverbs as labels.
- URL and emails: These are links to emails and web sites related to the theme or the entities; for example, Wikipedia references, YouTube videos or photographs.
- Phone numbers: These are combinations of digits referring to phone numbers. They are saved and rescued separately from the input sentence.
- Money expressions: These are the extraction of expressions related to currency and money in general; for instance, expressions that contain an utterance such as *50€* or *$15*.

Some researchers have evaluated the opinions of users about specific topics or products by analyzing the sentiment of their messages in social networks [45–47]. However, sentiment extraction fails, for example, when dealing with irony [48,49]. Irony is a characteristic of the human language that cannot be detected by state-of-the-art IEx algorithms.

The experiments show that it is possible to identify the proper nouns of the following categories: person, place, facility, event and organization. If the entity does not fit into any of these categories, the type is unknown. Using the following text as an example “Kobe Bryant is a basketball player with the Los Angeles Lakers. His next game is at Staples Center in Los Angeles”, the extracted entities were: Kobe Bryant (type: person), Los Angeles Lakers (type: organization), Staples Center (type: facilities) and Los Angeles (type: place). The concepts would be: “basketball”, “player” and “game”.

Since this module requires calling web services, the processing time depends on the bandwidth. Experiments show a mean time that is around one second.

#### 5.3.2. Information Enrichment

**Figure 6:**
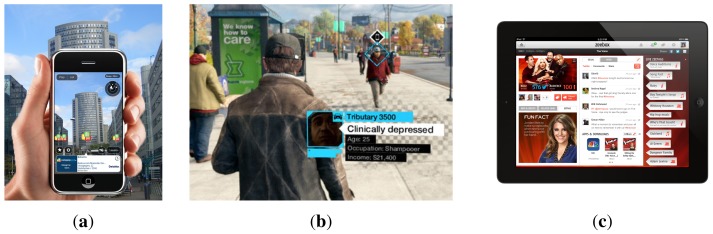
Layar, Watchdogs and augmented television. (**a**) Screen-shot of the Layar app. Additional information is superposed onto the image on the smart-phone. (**b**) Screen-shot of the game Watchdogs. In the game, enriched information is presented in the form of cards with details about characters or objects. (**c**) Zeebox is an augmented television app that shows live information about the current content.

IEn was first introduced by Salmen *et al.* [50]. They proposed IEn not by changing the data to which it is applied, but rather by adding an extra semantic layer to this data. Enriched information is getting very popular in IT fields. Companies are using this functionality with the intention of attracting new customers, retaining the old ones, increasing sales or surprising the users. A clear example is the rise of technologies, such as second screen or augmented television. These technologies give users the ability to complement media content with additional information. Some social TV services that use enriched information are Classora Augmented TV [51], Beamly/Zeboox [52] (Figure 6c), MioTV [53], GetGlue/Tvtags [54], Tockit [55] and Vuqio [56]. They can show the messages from social networks related to the content, the name of the actors in a film or what the content is about, for instance.

Another example of information contextualization is augmented reality. In this case, the user is moving in the environment and using specific devices (glasses, watches or smart-phones) that perceive additional information. As an example of this idea, Layar [57] is a popular augmented reality app for Google Glass [58] and smart-phones. By means of the camera embedded in the device, it lays digital content in real time over the user's line of sight (when wearing Google Glass) or over the image on the cell phone (Figure 6a). The digital content is classified in layers, and each layer offers different services (e.g., searching hot spots like restaurants or details about a landmark).

The gaming industry has also shown interest in enriched information. The popular game Watchdogs [59] is an example. In this game, the player can get extra information about the objects and other characters in the game, initially unknown, while they move in the virtual world (Figure 6b). This extra information is shown as “cards”, where the player can find the details about a character or the instructions for using an object. This information helps the player to progress within the game.

Contextualized dialogs are mainly applied to mobile robotics with different purposes, for instance acquiring and learning how the environment is semantically structured [17], learning new routes in a constrained map [60], resolving deictic orders [61] or in collaborative tasks where the robot has to take into account the user's point of view (see [62,63] and many others).

Lemaignan [64] presented his Ph.D. on grounding in HRI. He proposed a typology of desirable features for knowledge representation systems, supported this with an extensive review of the existing tools in the community and applied this to an HRI context. Lemaignan has released the open-source software called ORO (OpenRobots Ontology framework), compatible with the main robotics architectures (Robotic Operative System (ROS) and YARP). ORO provides several advanced reasoning services. Moreover, he has drafted a proposal for a standard API for knowledge manipulation that supports the specific needs of robotic applications.

Information enrichment consists of contextualizing some baseline information by adding extra knowledge. In this paper, the results of the information extraction (entities or concepts) can be used as the baseline information. In practice, information enrichment adds a description of an entity, a more precise type, or attaches news, videos or posts on social networks related to it. All of this extra information is obtained from knowledge databases, which relate an entity to information from the real world, as described in [65,66], and have been integrated into the ARDS.

In the previous example, the entity referred to as Kobe Bryant, which is of the type person, is enriched by including a more precise description: he is a basketball player, a Wikipedia entry (“an American professional basketball player for Los Angeles Lakers of the National Basketball Association”) and links to his official website, his Facebook account, his Tweets, the latest news about him or his YouTube channel.

Information enrichment results in knowledge that is framed within the pragmatic level in the natural language scheme (see Section 3).

There are several free access and on-line knowledge databases that are usually built by adding data typed by a programmer, that is with human intervention. The information enrichment module mainly uses Freebase [67], which is used in “Google's Knowledge Graph” to perform semantic searches [68]. Freebase gives a unique ID to each entity, which is used to find related information in other knowledge bases, for example film related data in IMDB, videos on YouTube or more detailed information from DBPediaused in the Wikipedia.

Currently, Google is working on replacing the Knowledge Graph based on Freebase with Knowledge Vault. In contrast to Knowledge Graph, which needs human intervention to increase the knowledge base, Knowledge Vault is automatically created as explained in [69]. This new knowledge base may be integrated in ARDS without much modification.

In some cases, Freebase can return ambiguous results. This is the case when Freebase connects an input entity (e.g., Madrid) with more than one output entity (e.g., “Real Madrid” and “City of Madrid”). In order to clearly identify the right entity, we query other knowledge bases, for example Classora [51] and Wolfram Alpha [70]. If it is certainly identified by any of the new knowledge bases, the system continues the information enrichment process. Otherwise, it uses the context of the message from which the entity has been extracted. Thus, following the previous example, if the topic is football, “City of Madrid” is discarded, and “Real Madrid” is selected as the first right entity related to the word “Madrid”. If, after this process, the ambiguity is still high, the input entity is automatically rejected.

The available new information added in the information enrichment process of an entity or concept includes the following fields:

- Full name: the input entity may not include the full name. For instance, if “Messi” is detected as an entity in the IEx process, it is passed to the IEn process, and it will return the full name, “Lionel Andrés Messi”.
- Subtype: a type that is more precise than the type provided by the IEx, e.g., “football player” or the city where the person was born and the country.
- A brief description of the entity or concept, as a paragraph.
- The most popular, high-resolution image of the entity or concept.
- The related article link from Wikipedia.
- Links to e-commerce web sites or app markets with content related to the entity, e.g., Google Shopping, Google Play, Amazon App Store, Ebay.
- Links to online music services: Spotify, Soundcloud, Grooveshark, Goear.
- Link to the official YouTube channel of the entity.
- Links to the entity's accounts on social networks, mainly Twitter and Facebook.
- Official web site of the concept, if it exists.
- Related news obtained from the Google News service.

Not all entities have all of the enriched information related to every field. For instance, some of the entities could not exist as a person or could not have an official YouTube channel, nor personal website.

Moreover, the IEn process is very slow considering that processes in robots should run in real-time. This is because the on-line provider services usually take a long time in returning the queried information. Experiments give a mean of 30 s when querying data from a new entity. In order to deal with this problem, we have developed a local cache memory that stores the enhanced information related to each entity the first time it is processed. Subsequent queries related to these entities, which are considered as familiar, do not need to make subsequent calls to obtain the enhanced information. This cache memory decreases the mean response time to milliseconds, which is in a suitable range for a real-time response. The cache memory uses the the MongoDB [71] database, characterized by its high-speed data access. This fact is particularly important when dealing with a high volume of data.

### 5.4. Timing Information of ARDS

**Figure 7:**
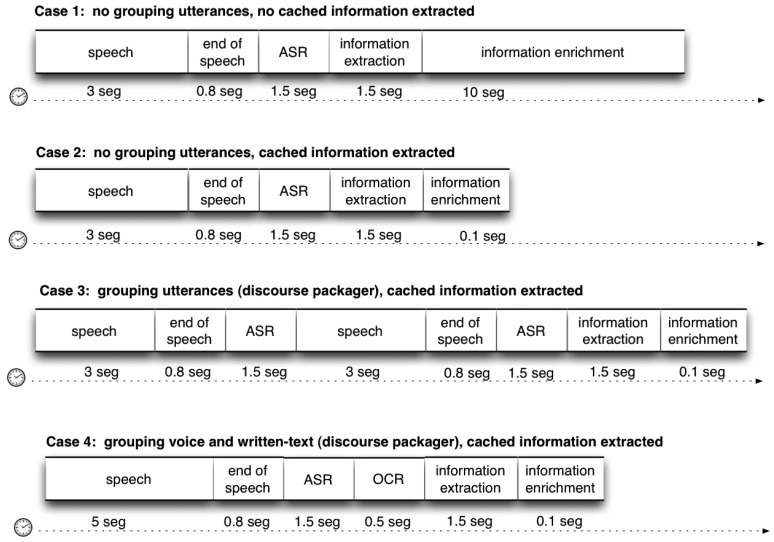
Timing information on several use cases. OCR, optical character recognition.

In Table 1, we summarize the time consumed for each ARDS component. Moreover, in Figure 7, we show the different use cases with different configurations and, therefore, different consumptions of time.

## 6. Proof of Concept: HRI and ARDS

In order to make the first test of how well the system is integrated within the rest of the main existing components of the robot, this section shows the first proof of concept: a user interacting by ARDS with the robot in a laboratory environment.

This proof of concept is focused on showing the reader how the extraction and enrichment of the information provided by a user during a human–robot dialog can improve HRI. The rest of the capabilities of the ARDS are out of the scope of this paper and have been presented in other previous publications, as shown above in the system description. We have deliberately kept the interactive skills as simple as possible with the intention of clearly focusing on the potential that ARDS offers with regard to its new features.

### 6.1. Scenario Description

The scenario is located in the laboratory, where a person interacts with Maggie by the ARDS. The capacities of the robot have been extended to use an external screen as a common user device, such as a tablet or a smart-phone. These devices are supported by the ARDS architecture and configured just as another output communicative modality, which shows how versatile the general architecture and the fission module are. The robot will present additional or enriched information on the screen according to the topic of the dialog, for instance the semantic website links obtained. These links are shown in the output devices in a common browser, so the user can interact with them as is usual when navigating the web. At the same time, the interaction with the robot is occurring. Examples of scenes of the system include:

- The user checks one of the received links and verbally asks the robot for more information while interacting with the content of the link: text info, picture, video or website.
- The user checks a received link and reacts with an emotional verbal or non-verbal response. The ADSR perceives that and orders the robot to express a similar emotion by multimodal gestures expressing laughing, surprise or interest (looking at the video).
- The user checks a received link and the robot makes some comment about the content, such as “oh! look at this!”, “isn't it nice?”, “this is pretty good!”, or asks the user about his/her opinion:
  “do you like it?”, “is that related to what you're talking about?”, “do you want to know more about that topic?”.

This scenario is chosen by the predefined dialog that is loaded in the dialog manager component. The DM is based on collaborative task theory [72] and has been used in other scenarios, as shown in [73], where a non-expert user programs the robot by interaction. The loaded dialog has several information slots that have to be filled in by means of interaction. For simplicity in the test, the interaction mode based on written text was not used, and in this proof of concept, these slots have just been filled by the user's speech modality. Other previous experiments had shown the success of such a mode, and as the multimodal fusion module abstracts the perceived information from its modality, using only voice as the input modality works fine for the proof of concept.

The user holds a wireless SingStart microphone in their hand and communicates with the robot using verbal dialog. On the other side, the robot responds using several modalities, such as voice, in the Spanish language, gestures and the tablet. The predefined information slots in the main dialog used as a proof of concept include the main theme of the conversation and the entities cited in speech; therefore, the main goal of the dialog is to complete these fields.

### 6.2. Implementation in Maggie and in a Visual External Device

The ARDS, as with the rest of the robotic software, has been implemented in Maggie using the Robotic Operative System (ROS) [74], which is a well-known framework for building and integrating robotic applications. The ARDS modules have been implemented as ROS nodes, and they communicate with each other by the ROS message passing system based on the so-called topics.

After the information enrichment module processes the incoming user input, which is going to be described below, the enriched information obtained can be used to fill information slots from a predefined dialog implementation (the DM is in charge of handling information to fill the slots with the right information perceived by the sensors; the information in the slots triggers new transitions in the dialog). Moreover, this enriched information is expressed to the user while the dialog is continuing the interaction, which is a novel contribution inside the HRI field.

Dialog transitions order the action of showing enriched information on the screen. To this end, the robot Maggie controls an Android tablet with which the user can interact. Thus, the enriched information is shown on the tablet concurrently with the execution of the dialog.

Each entity has its own card where all of the information obtained is presented. This card is split into different sections for the different kinds of information linked to the entity. There is one card for each entity, and the user can scroll up or down to explore the card, or scroll right or left to jump to another card. In this manner, the information shown in the tablet is related to the entities extracted from the user's messages during the interaction, so the enriched information is also shown in an interactive way.

**Figure 8:**
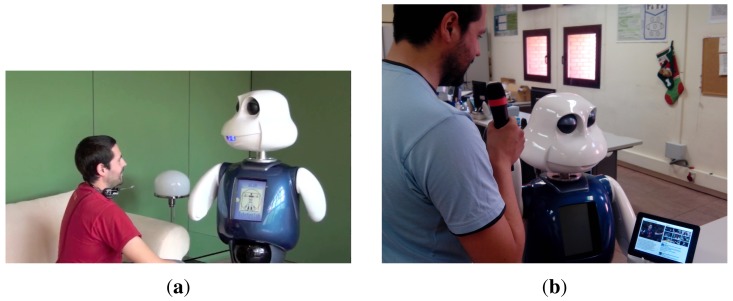
Human–robot dialog where the robot is showing information on a tablet about the main entities extracted from the conversation. (**a**) Maggie, a robotic platform for HRI research; (**b**) the remote tablet as an output modality.

Figure 8 shows the user and the robot interacting within the proof of concept in two different scenarios: in a common natural environment, a living room, and at the laboratory. Note how the robot is able to gaze at the external screen where the enriched information is shown, so a triangular interaction between the screen, the robot and the user is generated.

### 6.3. Data Flow in a Case Study

**Figure 9:**
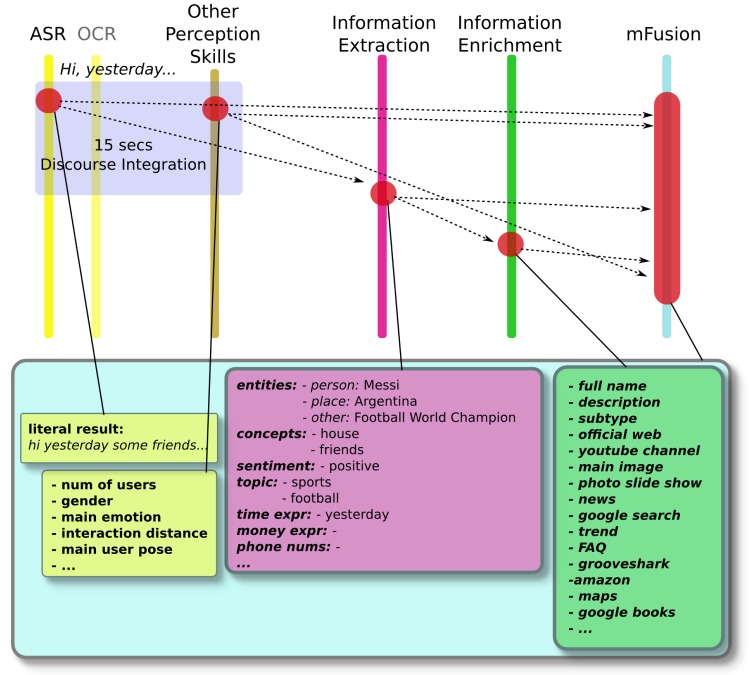
Information flow from user utterance to information enrichment.

In this section, we present, step by step, how information is computed during the dialog. The case study shows how the human's speech is used to extract the semantic content, main entities and concepts, which later will be semantically enriched with information obtained from the cloud. As shown in Figure 9, the data flow follows different processes: open-grammar ASR, information extraction, information enrichment, multimodal fusion, DM moves and multimodal expression.

The following sections explain what each process does.

#### 6.3.1. Speech Recognition and Information Extraction

The robot starts talking, and the user formulates a common statement:

MAGGIE: Hi, David. What have you been doing lately?

USER: Hi! Yesterday, some friends and I were watching the Football World Cup at my house. Argentina's game was great, but Messi did not play well.

[*Now, the system will analyze the user utterance semantically.]*

The discourse packager module (see Figure 4) concatenates all of the utterances received within a time frame of about 15 s if there is no more than one second of silence. This period value has been chosen empirically after several previous tests. Afterward, the concatenated sentence is sent out to the IEx module. The process of IEx is based on the external service TextAlytics, which has been completely integrated into the system. The extracted entities from the IEx module have been completely contextualized in the IEn process. The rest of the extra information, such as concepts, type, topic or sentiment, could also be contextualized in the same way.

The ARDS then recognizes the user's statement and returns the following three sentences:

- (i) “Hi”
- (ii) “Yesterday, some friends and I were watching Football World Cup at my house”
- (iii) “Argentina's game was great, but Messi didn't play well”

Notice that the utterances recognized by the open-grammar ASR are almost literal. In this experiment, we have not used any written text, so the OCR module has not recognized any text, and it did not return any result. Since the discourse packager uses a time frame of 15 s, the three sentences are easily integrated in a concatenated unique utterance:

- “Hi Yesterday some friends I were watching Football World Cup at my house Argentina's game was great but Messi didn't play well”

If the OCR mode were also used, at this stage, the recognized text transcription would also be concatenated at the end of the string by the discourse packager.

The IEx module analyzes the sentence, so as to structure the information conveyed. The entities returned by this module can be checked out through the service website. So, the results obtained by the TextAlytics service is shown in Table 2.

The entities are enumerated according to their types: person, place, other. Notice that the sentiment is acquired from the semantic information of the input text and not by the suprasegmental features of the audio of the user speech.

#### 6.3.2. Information Enrichment and Multimodal Fusion

As explained before, for this proof of concept, the IEn is performed considering only the entities that have been identified, and not with the rest of the information the module gives. Following the algorithm presented in Section 5.3.2, the enriched information related to each entity is extracted. All of this new information is shown in Table 3. Note that URLs have been shortened, and they are clickable.

Figure 9 shows how the multimodal fusion process is performed. Notice that both the information extraction and the information enrichment modules are in execution concurrently with the other perception skills. For instance, there is a skill that extracts some features of the user, analyzing the voice footprints, such as his/her name, gender [22], or the main emotion, using multimodal information [21]; also, there is another skill that localizes the external sound source in space [32], so it gives 2D information about where the user is. The multimodal fusion module groups together the enriched information with the data provided by the other perception skills [24]. All of the packed information is sent to the DM. Therefore, the IEx and the IEn modules work as meta-perception skills, as the information they provide is also fused with other perception skills. This aspect of multimodal fusion is important, since it allows the DM to decide on different ways of showing the enriched content, depending on the gender of the user, his/her main emotional state and his/her position with respect to the robot.

The multimodal information package that includes data from the perception skills and the IEx module is as follows:

Number of users: one
Gender of user: male
Main user emotion: tranquility
Interaction distance: space 2 (1.20 m)
Pose: standing and gazing
Thematic: sports, football
Sentiment: positive
Entities: Messi (person), Football World Champion (other), Argentina (place)
Concepts: house, friends
Time expressions: yesterday

Additionally, it also includes all of the semantic information gathered by the IEn module. This multimodal package has been called the communication act and is the main input data for the DM that handles the interaction loop with the user.

#### 6.3.3. Dialog Manager and Multimodal Expression

The DM receives the packaged data as input, evaluates it and performs the consequent dialog moves, executing the action programmed in the dialog plan. The incoming information is used to fill different information slots that are defined in the dialog. During this proof of concept, such slots are filled with the inferred entities, and the dialog orders expressing the enhanced information related to those entities to the user.

**Figure 10:**
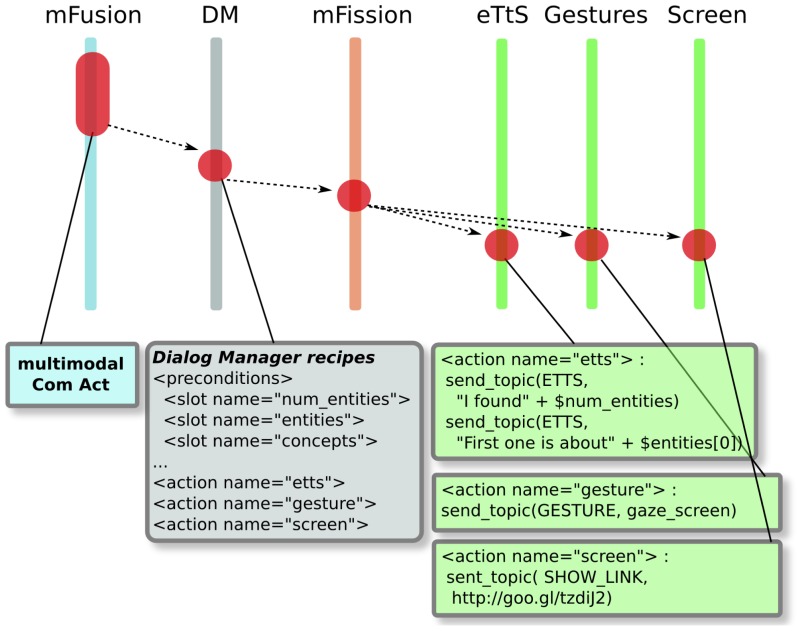
Information flow from multimodal fusion to multimodal expression.

Figure 10 shows the information flow from the perceived communication act (CA) to the robot's multimodal expression. The fusion module sends the CA to the DM, which fills some of the information slots defined in the active dialog. These slots include what the information extraction module has detected: the number and names of the main entities and concepts and also the IEn data: URLs related to such entities.

**Figure 11:**
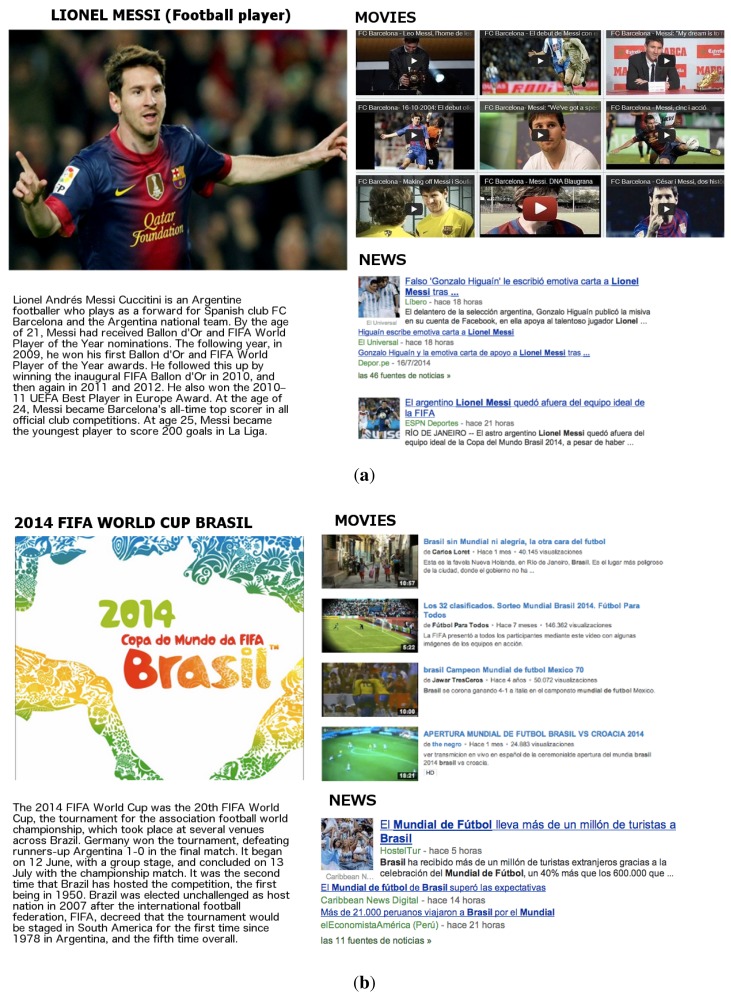

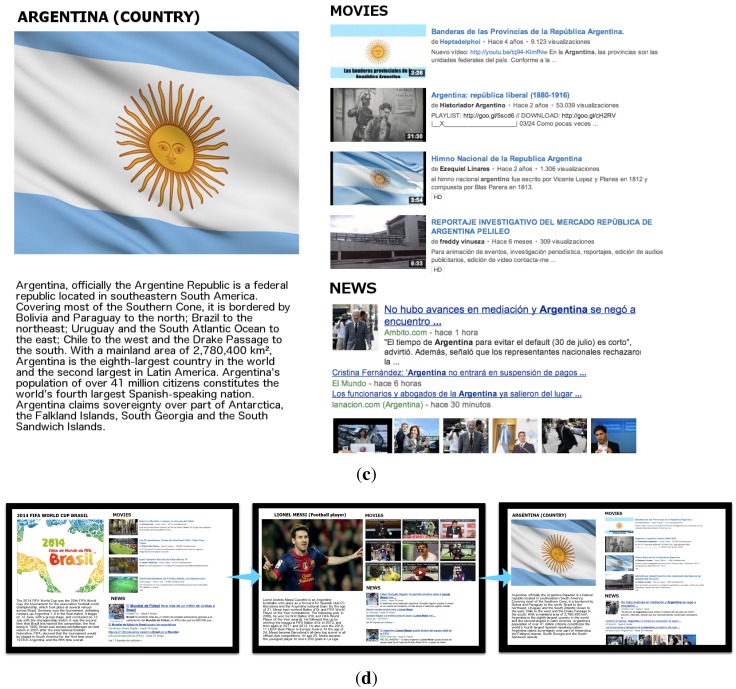
Entity cards shown on the tablet to the user. (**a**) Messi; (**b**) Football World Cup; (**c**) Argentina; (**d**) transitions between entity cards.

In the present case, the dialog just makes an echo or confirmation of what has been detected and shows the enriched information on the external screen. This is made by sending an expressive communicative act to the multimodal fission module. This module takes charge of synchronizing the expression in the different modalities: gestures, speech and the visual mode in the external screen. Thus, the dialog is designed in such a way that when an information slot in the dialog is filled, the robot communicates additional information about the entity detected. The multimodal fission module decides how to communicate this additional information. In this case, it uses the verbal mode to verbalize the description of the entities and the visual mode to show the related obtained cards in the screen of the external tablet (Figure 11).

Notice that the dialog loaded in the DM module could include more complex sub-dialogs, such as confirmations, comments or appreciations. The main contribution of the system is that is able to show augmented information while the robot keeps on interacting with the user and incorporates such information into the interaction process itself.

## 7. Conclusions and Future Research

This paper has described the augmented robotic dialog system and its implementation in the social robot Maggie. Other similar research has already used natural language processing techniques, IEx feature, and IEn to improve the user experience, but none in a unique, complete system, nor in an interactive social robot.

One of the main advantages of the ARDS is the possibility of communicating with the robot both with or without a grammar, that is using natural language. Grammars are formed by rules that delimit the acceptable sentences for a dialog. The use of grammars allows the dialog system to achieve a high recognition accuracy. On the other hand, grammars limit considerably the interpretable input language. The use of a grammarless ASR in conjunction with the IEx modules enables interacting with the robot using natural language. These modules process the user utterances and extract their semantic information from any natural input utterance. Later, this information is used in the dialog, showing related multimedia content.

In addition, the ARDS facilitates maintaining a coherent dialog. The main topics of the dialog can be extracted; thus, the robot can keep talking about the same matter or detect when the topic changes.

Encouraging pro-active human–robot dialogs is also a key point. The IEn module provides new related information about what the user is talking about. The robot can take the initiative and introduce this new information in the dialog, driving the dialog to new areas while coherence is kept.

Although it could be also applied to other areas, it is important to remember that the ARDS has been designed for social robots, which are robots intended mainly for HRI. For these robots, it is important to engage people in the interaction loop. Considering the strong points already mentioned (natural language understanding, coherence and proactive dialogs), the ARDS tries to improve this engagement.

Moreover, the sentiment extracted from the user's message can help to improve engagement, as well. It can be used to detect when the user is losing interest in the conversation, so the robot can try to recover in this situation. Besides, the robot's expressiveness is complemented with unconventional output modes, such as a tablet, which can make the result more appealing to users.

As we stated in the Introduction, our final aim is to ease the HRI, making it more human-like. The presented dialog system will be tested by non-expert users freely interacting with the robot. In particular, we are especially interested in patients, children, the elderly and people suffering from cognitive disorders (like attention deficit or memory disorders). We believe that these groups can benefit the most, but also, they are the most difficult to interact with, due to their limitations. In fact, one of the first benchmarks for the ARDS will be a group of Alzheimer's disease patients, where a social assistant robot supports them in their daily tasks, as shown in [75].

## Figures and Tables

**Table 1:** Time comsumption of ARDS's components.

**ASR Transcription**	Consider end of voice activity: 800 ms.
	Voice to text transcription by web-service: 1.5 s.

**OCR Transcription**	500 ms

**Discourse Packager**	Could be tuned between 0–60 s.

**Information Extraction**	1.5 s.

**Information Enrichment**	For unknown entities can take to process for each of them between 10–30 s.
	For cached entities, less than 100 ms.

**Table 2:** Results obtained by the Information Extraction module from the user utterance: *Hi! Yesterday, some friends and I were watching the Football World Cup at my house. Argentina's game was great but Messi didn't play well*.

**Entities (type)**	Messi (person),
	Football World Champion (other)
	Argentina (place)

**Concepts**	house, friends

**Sentiment**	positive

**Topic**	sports, football

**Time Expressions**	yesterday

**Money Expressions**	-

**Telephone Numbers**	-

**Table 3:** Results obtained by the Information Enrichment module for the user utterance: *Hi! Yesterday, some friends and I were watching the Football World Cup at my house. Argentina's game was great but Messi didn't play well*.

**Name**	**Football World Cup**	Messi	Argentina
**Full Name**	Football World Cup Brazil 2014	Lionel Messi	Argentina
**Description**	The 2014 FIFA World Cup was the 20th FIFA World Cup, the tournament for the association football world championship, which took place […]	Lionel Andrés Messi Cuccitini is an Argentine footballer who plays as a forward for Spanish club FC Barcelona and the Argentina national team […]	Argentina, officially the Argentine Republic is a federal republic located in southeastern South America. Covering most of the Southern Cone […]
**Subtype**	Tournament	Football player	Country
**Official Web**	http://goo.gl/tzdiJ2	http://goo.gl/bivGsX	http://goo.gl/gxsf0g
**YouTube Official Channel**	http://goo.gl/EYcbYO	http://goo.gl/mKP0wD	
**Main Image**	http://goo.gl/GaOSnA	http://goo.gl/Qxtgft	http://goo.gl/bwKjGI
**Photo Slide Show**	http://goo.gl/M3MVbB	http://goo.gl/Ao0xta	http://goo.gl/M8CynZ
**News**	http://goo.gl/fTrO47	http://goo.gl/OlGq1N	http://goo.gl/FQ9Lac
**Google Search**	http://goo.gl/HF0xYS	http://goo.gl/6UHx0T	http://goo.gl/x4gXzR
**Trend**	http://goo.gl/ctHmyD	http://goo.gl/ns2omR	http://goo.gl/A4uHHu
**Images**	http://goo.gl/Q0Wbfu	http://goo.gl/FzwTa7	http://goo.gl/Y5gJ9p
**YouTube Search**	http://goo.gl/8mkj4n	http://goo.gl/xupCUC	http://goo.gl/kQ0q0w
**FAQ**	http://goo.gl/rgnqde	http://goo.gl/YbP1U6	http://goo.gl/iKWJjv
**Grooveshark**	http://goo.gl/7ruSv2	http://goo.gl/t0l1qr	http://goo.gl/bpGgT4
**Soundcloud**	http://goo.gl/hDMbVf	http://goo.gl/ekrGwX	http://goo.gl/z8lyQa
**Amazon Music**	http://goo.gl/B0pBs1	http://goo.gl/90Akzb	http://goo.gl/j1HNhd
**Spotify**			http://goo.gl/5yr1Wi
**Goear**		http://goo.gl/fcFYLt	
**Google Books**	http://goo.gl/G6cPWt	http://goo.gl/mglsAo	http://goo.gl/2rfGzy
**Map**			http://goo.gl/TMSPfd
**Atrápalo**			http://goo.gl/5TRky3
